# Metabolism-Related Signature Analysis Uncovers the Prognostic and Immunotherapeutic Characteristics of Renal Cell Carcinoma

**DOI:** 10.3389/fmolb.2022.837145

**Published:** 2022-03-28

**Authors:** Jianye Zhang, Qi Zhang, Yue Shi, Ping Wang, Yanqing Gong, Shiming He, Zhihua Li, Ninghan Feng, Yang Wang, Peng Jiang, Weimin Ci, Xuesong Li, Liqun Zhou

**Affiliations:** ^1^ Department of Urology, Peking University First Hospital, Beijing, China; ^2^ Key Laboratory of Genomics and Precision Medicine, Beijing Institute of Genomics, Chinese Academy of Sciences, Beijing, China; ^3^ Institute of Urology, Peking University, Beijing, China; ^4^ National Urological Cancer Center, Beijing, China; ^5^ Urogenital Diseases (Male) Molecular Diagnosis and Treatment Centre, Peking University, Beijing, China; ^6^ Department of Urology, Affiliated Wuxi No. 2 Hospital of Nanjing Medical University, Wuxi, China

**Keywords:** renal cell carcinoma, metabolism, MRPScore, prognosis, immunotherapy

## Abstract

Renal cell carcinoma (RCC) is one of the most common urological cancers. RCC has a poor prognosis and is considered a metabolic disease. It has been reported that many metabolic pathways are associated with the development of RCC. However, the prognostic value of metabolism-related genes in RCC is unclear. We herein aimed to establish a scoring system based on the gene expression profile of metabolic genes to evaluate the response to immunotherapy and predict the prognosis of RCC. In this study, we collected multicentre RCC data and performed integrated analysis to characterize the role of tumour metabolism in RCC and explore the relationship between metabolism and prognosis and immune therapy. Based on transcriptomic data, metabolism-related genes were used for nonnegative matrix factorization clustering. We obtained three subclasses of RCC (M1, M2, and M3), and they are associated with different prognoses and immune infiltrate levels. Then, based on the pathway activity of 113 metabolism-related gene signatures, we classified patients into three distinct metabolism-related signatures. Finally, we provide a metabolism-related pathway score (MRPScore) that is significantly associated with RCC prognosis and the response to immunotherapy. Taken together, in this study, we established an RCC classification system based on metabolic gene expression profiles that could further the understanding of the diversity of RCC. We also present the MRPScore, which may be used as an indicator to predict the response to clinical immune therapy.

## Introduction

Renal cell carcinoma (RCC) is one of the most common urological malignancies. Globally, it is estimated that there were 431,200 new cases and 179,400 RCC-related deaths in 2020 (Sung et al., 2021). The incidence rate of RCC has been steadily increasing by 2–4% each year (Hsieh et al., 2017). Clear cell RCC (ccRCC) is the main subtype of RCC, and it accounts for approximately 75% of all RCCs (Moch et al., 2016). Approximately 30% of patients with ccRCC have developed distant metastases at the time of diagnosis, (Xue et al., 2019), and 90% of RCC patients die from tumour-specific recurrence and metastasis (Siegel et al., 2019). RCC patients are insensitive to radiation, so radical nephrectomy or multitarget tyrosine kinase inhibitors (TKIs) and mammalian target of rapamycin (mTOR) inhibitors are the major treatment modalities for localized and metastatic RCC patients. Some RCC patients may benefit from PD-1/PD-L1 blockade immunotherapy; however, the responses to immunotherapy of patients are variable (Gill et al., 2017; McKay et al., 2018).

It has been reported that dysregulated cellular metabolism contributes to the development and progression of RCC. RCC is characterized by increased consumption of glucose and simultaneous enhanced production of lactate under normal oxygen supply (the Warburg effect). The other metabolic features of RCC include alterations in the tricarboxylic acid cycle (TCA), the pentose-phosphate pathway and the metabolic imbalance of amino acids and fatty acids (Wettersten et al., 2017). Morphologically, ccRCC cells are lipid- and glycogen-laden, (Gebhard et al., 1987), indicating that altered fatty acid and glucose metabolism may play a key role in the development of ccRCC. Loss of VHL leads to aberrant accumulation of HIFα, which results in uncontrolled activation of HIFα target genes that regulate angiogenesis, glycolysis, and apoptosis (Majmundar et al., 2010; Semenza, 2013). It has been reported that the worse survival of RCC patients is correlated with the upregulation of the pentose phosphate pathway and fatty acid synthesis pathway genes and the downregulation of TCA cycle genes (Cancer Genome Atlas Research, 2013; Hakimi et al., 2013). Therefore, the development of RCC could be associated with many metabolic pathways and genes rather than specific ones. However, there is no systemic integration between metabolism-association patterns, prognosis and immunotherapy.

In this study, according to different metabolism-associated gene signatures, we identified three patterns in RCC that showed a correlation with molecular and clinical characteristics. The three metabolic patterns were associated with overall survival (OS), progression-free survival (PFS) and tumour microenvironment (TME) features. From the metabolism-association pathways, we obtained three metabolic clusters that were significantly correlated with three metabolic patterns and could predict the prognosis of RCC patients. Finally, we built a scoring system named MRPScore to quantify the metabolic status based on the metabolism-related pathway signature. The MRPScore may act as an indicator of prognosis, immune infiltration, and immunotherapy response in RCC. More importantly, a scoring system is more intuitive and practicable for clinical application.

## Materials and Methods

### Patient Cohort

Multiple data repositories, including the TCGA database from the Xena browser (GDC hub: https://gdc.xenahubs.net), were used to collect the available clinical information of cancer patients. This included information regarding the age, survival status, tumour grades, tumour stages, T stage (T) status, metastasis (M) status and RNA-seq data from 530 kidney renal clear cell carcinoma (KIRC) and kidney renal papillary cell carcinoma (KIRP) patients. RNA-seq data were obtained to analyse the transcriptome profile of RNA expression and were measured using fragments per kilobase of exon per million fragments mapped (FPKM). We performed a log2-based transformation to normalize the RNA expression profiles. For datasets in the CPTAC dataset, the RNA sequencing data (reads per kilobase per million mapped reads---RPKM) of gene expression and clinical data of 110 renal tumour samples were downloaded from https://proteomics.cancer.gov/programs/cptac (Integrated Proteogenomic Characterization of Clear Cell Renal Cell Carcinoma). The processed data for the dataset from the E-MTAB-1980 cohorts were downloaded from the website (https://www.ebi.ac.uk/arrayexpress/experiments/E-MTAB-1980/). (Sato et al., 2013) For the immunotherapy cohorts, we included the processed gene expression of a metastatic urothelial cancer (mUC) cohort that received atezolizumab treatment by using the R package IMvigor210CoreBiologies (http://research-pub.gene.com/IMvigor210CoreBiologies) (Mariathasan et al., 2018) In addition, we obtained the mRNA expression of a pretreatment melanoma cohort (GSE78220) that underwent anti-PD-1 immune checkpoint inhibition (ICI) therapy from GEO (Hugo et al., 2016). We also obtained processed RNA-seq data in a transcript per million (TPM) matrix of patients treated with anti-PD1 ICI from a large melanoma genome sequencing project (MGSP) (Liu et al., 2019).

### Unsupervised Clustering for 113 Metabolism-Related Gene Signatures

We downloaded a comprehensive list of 113 metabolism-related gene signatures from previous research. (Yang et al., 2020). These gene sets cover a diverse range of functions in metabolism-related pathways, including amino acid metabolism-related signatures, such as glycine, serine and threonine metabolism, histidine metabolism, tyrosine metabolism and the urea cycle. It also included lipid metabolism-related signatures, such as fatty acid degradation, linoleic acid metabolism, retinol metabolism and steroid hormone metabolism and drug metabolism-related signatures, such as drug metabolism by cytochrome P450, drug metabolism by other enzymes and metabolism of xenobiotics by cytochrome P450. Unsupervised clustering analysis was employed to identify distinct metabolism-related patterns based on the activity of 113 metabolism-related gene signatures and classify patients for further analysis. The number of clusters and their stability were determined by the consensus clustering algorithm and k-means method. We used the ConsensuClusterPlus R package to perform the above steps, and 1,000 repetitions were conducted to guarantee the stability of classification.

### Gene Set Variation Analysis

Gene set variation analysis (GSVA) is a nonparametric and unsupervised gene set enrichment method that can estimate the score of certain pathways or signatures based on transcriptomic data (GSVA: gene set variation analysis for microarray and RNA-seq data). The 113 metabolism-related gene signatures were obtained from previously published studies, and the gene sets “c5. go.bp.v7.4. symbols” and “h.all.v7.4. symbols” were downloaded from the Molecular Signatures Database (MSigDB) and by running the GSVA R package. Subsequently, differential analysis was conducted based on the pathway activity scores using the limma R package in R software. Signatures with an absolute log2-fold change (FC) > 0.25 (adjusted *p* < 0.05) were considered significantly differentially expressed signatures.

### Estimation of Immune Infiltration

The microenvironment cell population counter (MCP-counter) is a methodology based on gene expression profile data. The MCP-counter was used to evaluate the absolute abundance of eight immune populations, including T cells, CD8^+^ T cells, natural killer cells, cytotoxic lymphocytes, B cell lineage cells, monocytic lineage cells, myeloid dendritic cells, and neutrophils, and two nonimmune stromal cell populations, including endothelial cells and fibroblasts. (Becht et al., 2016).

In addition, immune scores and stromal scores were calculated by using the ESTIMATE algorithm, which can reflect the enrichment of stromal and immune cell gene signatures. (Yoshihara et al., 2013).

### Identification of Differentially Expressed Pathways Between Distinct Metabolism-Related Phenotypes

To clarify metabolism-related phenotypes, we classified patients into three distinct metabolism-related phenotypes based on the pathway activity of 113 metabolism-related gene signatures. The empirical Bayesian approach of the limma R package was applied to determine the differential pathways between different metabolism patterns. The significance criteria for determining significantly differentially expressed pathways was set as an absolute log2-fold change (FC) > 0.25 (adjusted *p* < 0.05).

### Generation of Metabolic Gene Signatures

To quantify the metabolism-related patterns of individual patients, we constructed a set of scoring systems to evaluate the metabolic pattern of individual tumours with KIRC. This system is the metabolism-related pathway signature, and we termed it the MRPScore. The procedures for establishing the metabolism-related pathway signature were as follows: The differentially expressed pathways identified from different metabolism patterns were first normalized among all TCGA-KIRC samples, and the overlapping differentially expressed pathways were extracted. The patients were classified into several groups for deeper analysis by adopting an unsupervised clustering method for analysing overlapping differential pathways. The consensus clustering algorithm was utilized to define the number of pathway clusters as well as their stability. Then, we performed prognostic analysis for each pathway in the signature using a univariate Cox regression model. The pathways with a significant prognosis were extracted for further analysis. Then, a principal component analysis (PCA) was performed, and the principal component 1 was extracted to serve as the signature score by referring to a method similar to the TMEscore. (Zeng et al., 2019).

$$MRPScore=\sum PC1_{i}$$

where i is the activity of metabolism phenotype-related pathways.

### Correlation Between Metabolic Phenotype-Related Signatures and Other Related Biological Processes

Mariathasan et al. constructed a set of gene sets that stored genes associated with some biological processes, including CD8 T effector signatures, immune checkpoints, DNA damage repair, mismatch repair, nucleotide excision repair, DNA replication and antigen processing and presentation. (Mariathasan et al., 2018). We performed a correlation analysis to further investigate the association between metabolism-related signatures and some related biological pathways.

### Prediction of the Response to Immune Checkpoint Inhibitor Therapy

Based on tumour pretreatment expression profiles, the Tumour Immune Dysfunction and Exclusion (TIDE) module can estimate multiple published transcriptomic biomarkers to predict patient response. We employed the TIDE algorithm to predict the clinical response to ICI therapy of KIRC patients with default parameters. Patients with high TIDE scores were predicted to be nonresponders, while patients with low TIDE scores were considered to be responders.

### Statistical Analysis

All computational and statistical analyses were performed in R 4.0.2 software. Unpaired Student’s *t* test was used to compare two groups with normally distributed variables, while the Mann-Whitney *U* test was used to compare two groups with nonnormally distributed variables. For comparisons of three or more groups, Kruskal–Wallis tests were used. Survival analysis was carried out using Kaplan-Meier methods and compared by the log-rank test. A univariate Cox proportional hazards regression model was used to estimate the hazard ratios for univariate analyses, and a multivariable Cox proportional hazards regression model was used to estimate the hazard ratios for multivariable analyses. A two-tailed *p* value < 0.05 was considered statistically significant.

## Results

### Identification of Subclasses in RCC and Correlation of Three RCC Subclasses With Metabolism-Associated Signatures

**FIGURE 1:**
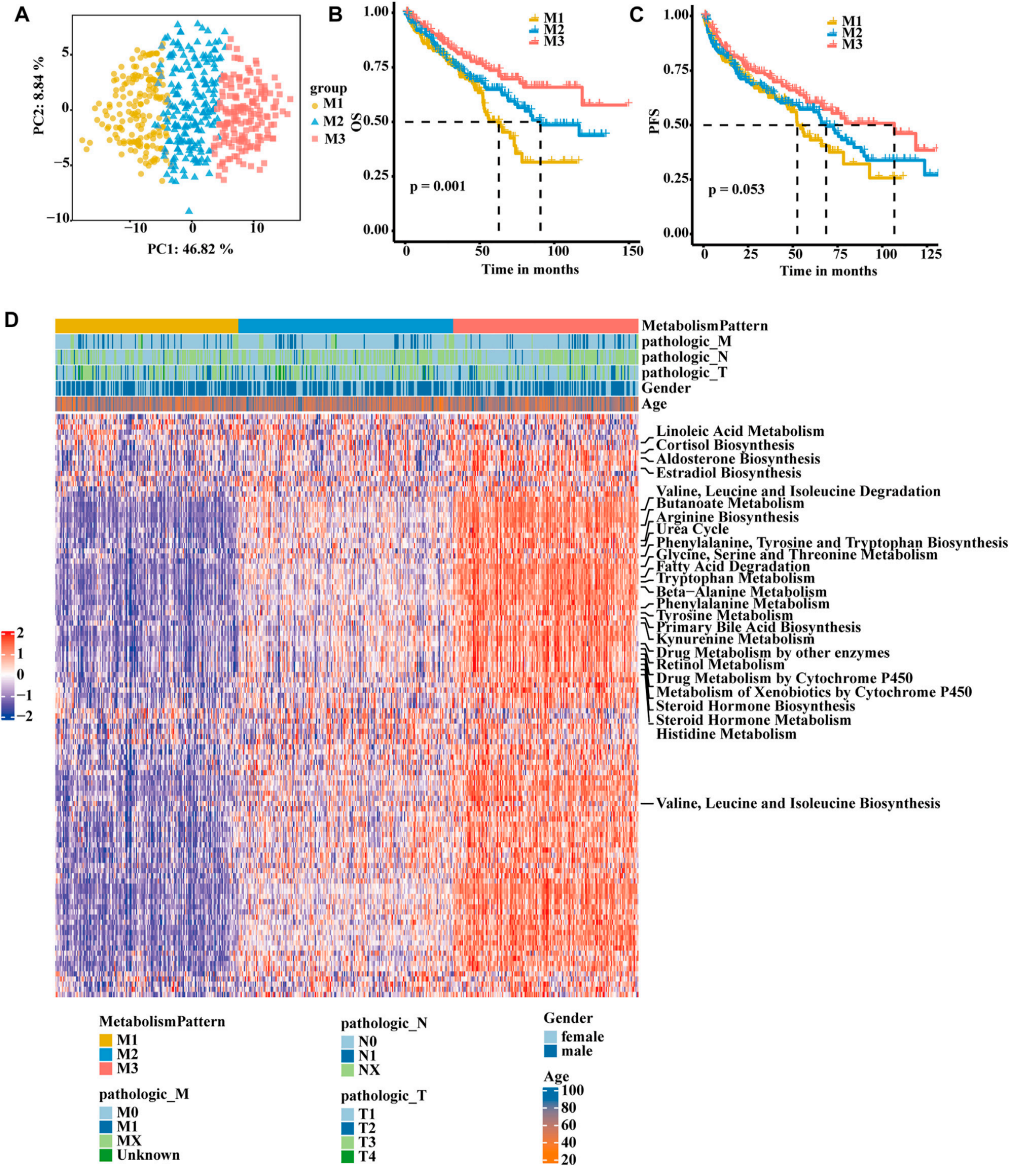
Identification of RCC subclasses according to metabolism-associated signatures in the TCGA-KIRC cohort. **(A)** Principal component analysis of the pathway activity of three metabolism-related patterns, showing a remarkable difference in pathway activity between the different metabolism patterns. **(B)** OS analyses for the three metabolism-related patterns based on patients with KIRC from TCGA cohorts. **(C)** PFS survival analyses for the three metabolism-related patterns based on patients with KIRC from TCGA cohorts. **(D)** Consensus clustering of 113 metabolism-related gene signatures between the three metabolism-related clusters in the TCGA-KIRC cohort.

A flow chart was developed to systematically describe our study (Supplementary Figure S1A). Based on consensus clustering of the expression profiles of the 113 metabolism-related gene signatures in the TCGA-KIRC cohort and E-MTAB-1980 cohort, we calculated and determined the optimal k value. Ultimately, k = 3 was chosen as the optimal number of clusters after comprehensive consideration (Supplementary Table S1; Supplementary Figures S1B–D). We identified three metabolism-related patterns according to principal component analysis for the activity of the pathway and named them M1, M2, and M3 (Figure 1A). Subsequently, we performed another independent analysis based on the E-MTAB-1980 cohorts with 101 RCC samples, and the results also showed that there were three metabolic subclasses of RCC (Supplementary Figures S2A–D). In the TCGA-KIRC cohort, patients in the M3 group showed a better prognosis, patients in the M1 group experienced a much poorer prognosis, and patients in the M2 group exhibited an intermediate prognosis (Figure 1B). PFS showed the same trend, although a significant difference was not observed (Figure 1C). In the E-MTAB-1980 cohort, the same result was obtained. The OS of M1 was significantly poorer than that of the other groups (Supplementary Figure S2E). Next, we further explored whether distinct subclasses had different metabolic characteristics. In the heatmap, the results showed that M2 and M3 had activated specific metabolic signatures, while M1 had no activity of specific metabolic signatures (Figure 1D). The activation pattern was the same in the E-MTAB-1980 cohort (Supplementary Figure S2F).

### Association Between Metabolic Patterns and the Molecular Characteristics of RCC

**FIGURE 2:**
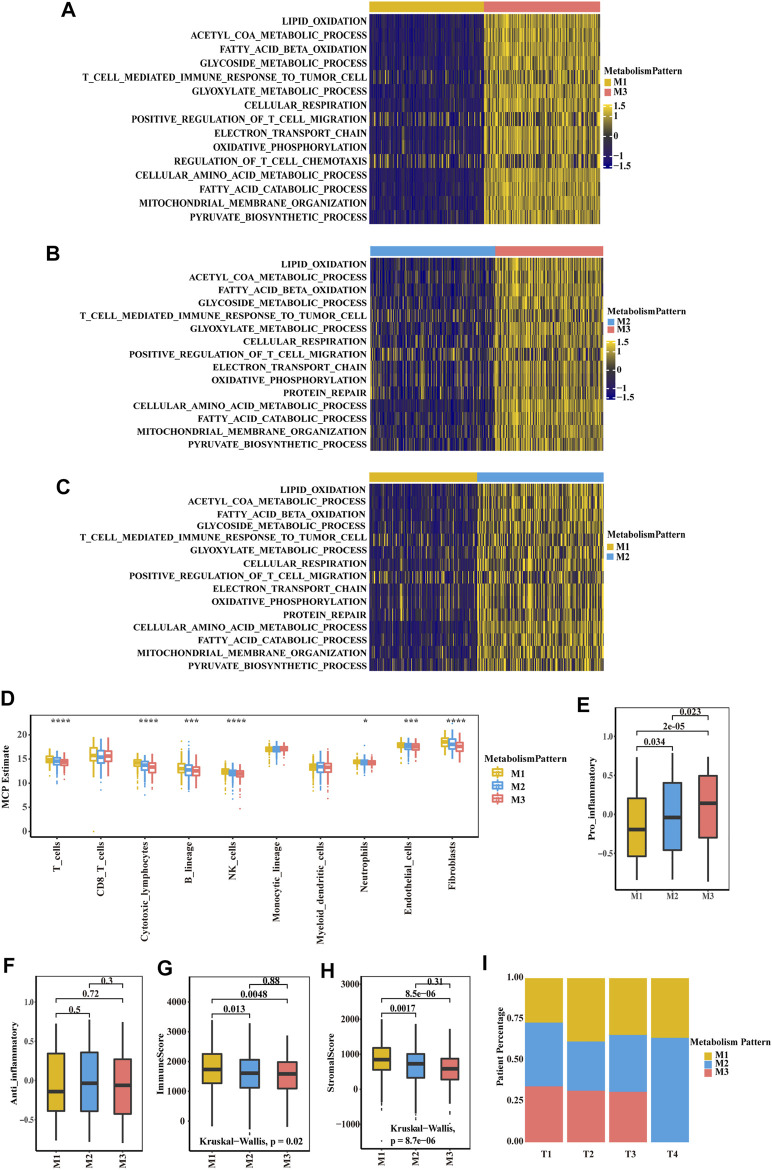
Biological characteristics of three metabolism-associated patterns in the TCGA-KIRC cohort. **(A–C)** GSVA enrichment analysis showing the activation states of biological pathways in distinct metabolic patterns. A heatmap was used to visualize these biological processes. Yellow represents activated pathways, and blue represents inhibited pathways. **(A)** M1 vs. M3 **(B)** M2 vs. M3 **(C)** M1 vs. M2. **(D)** Boxplot of the abundance of immune and stromal cell populations distinguished by different subclasses. The asterisks represent a statistically significant *p* value (*p < 0.05; **p < 0.01; ***p < 0.001; ****p < 0.0001). (**E–F)** Boxplot of pro-inflammatory macrophage **(E)** and anti-inflammatory macrophage **(F)** to compare the difference of three subclasses. *p* values were determined by a student’s *t* test. **(G-H)** Boxplot of immune score **(G)** and stromal score **(H)** from ESTIMATE of three subclasses. The Kruskal–Wallis test was used to compare the significant differences between the three subclasses. **(I)** The proportion of the three metabolic patterns in the different T stages (T), T1, T2, T3 and T4.

To further explore the biological function of the three metabolic patterns, we performed GSVA. Compared to M1 and M2, M3 showed enrichment in pathways associated with lipid oxidation, fatty acid beta oxidation, the glycoside metabolic process, cellular respiration, oxidative phosphorylation and the pyruvate biosynthetic process. However, M1 showed enrichment in metabolic silencing (Figures 2A–C; Supplementary Figure S3A). Thereafter, we deconvoluted immune cells and stromal cells in the TME and found that the M1 group had a higher composition of immune cells and stromal cells than the other two groups (Supplementary Table S32; Figure 2D). In the E-MTAB-1980 cohort, there were also more T cells, monocytes and stromal cells in the M1 group (Supplementary Table S3; Supplementary Figure S3B). Next, we focus on the two distinct macrophage phenotypes (pro-inflammatory and anti-inflammatory macrophages) contribute to the three metabolic patterns, we found that pro-inflammatory macrophages in M1 group was lower composition than M2 and M3, and there was no significant difference of anti-inflammatory macrophage in three patterns (Figures 2E,F). We then correlated the classification with the immune score and stromal score (Figures 2G,H; Supplementary Figures S3C,D). A significant difference was observed, with a higher median immune score and stromal score for M1 than for M2 and M3. After analysing the relationship between the clinical TNM stage and classification, it is interesting that the patients with a higher T stage were associated with more M1 group and less M3 group (Figure 2I; Supplementary Figure S3F). Patients in the less M3 group and more M1 group experienced more events of lymph node or distant tumour metastasis (Supplementary Figures S3E,G).

### Classification of RCC Subtypes by Differential Metabolism-Associated Pathways

**FIGURE 3:**
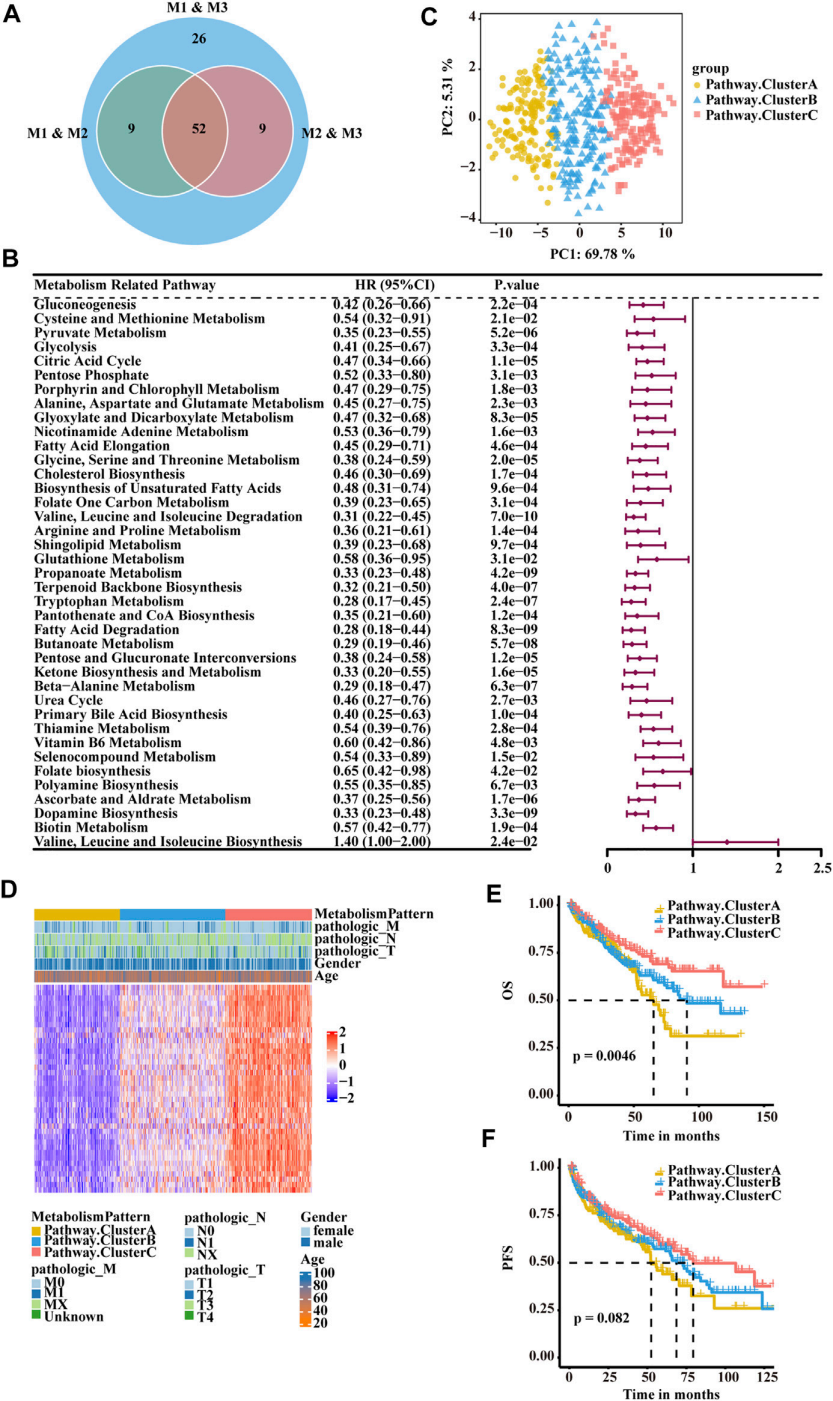
Classification of RCC clusters based on differential metabolism-associated pathways. **(A)** Venn diagram illustrating the number of differential pathways among the three metabolism patterns. **(B)** Forest plots showing the significantly prognostic differential pathways identified with univariate Cox regression analysis based on OS. **(C)** Principal component analysis of the pathway activity of the three pathway clusters, showing a remarkable difference in pathway activity between the different clusters. **(D)** Consensus clustering of prognostic differential pathways between the three pathway clusters in TCGA-KIRC. **(E)** OS analyses for the three pathway clusters based on patients with KIRC from TCGA cohorts. **(F)** PFS survival analyses for the three pathway clusters based on patients with KIRC from TCGA cohorts.

To quantify the metabolic patterns in RCC, we identified 52 differential pathways among the three metabolic patterns (Figure 3A). Univariate Cox regression was applied for the screening of the 52 differential pathways, resulting in 39 candidates that were significantly prognostic (Supplementary Table S4; Figure 3B). Principal component analysis for the pathway activity of the three pathway clusters showed a remarkable difference in pathway activity between different clusters (Figure 3C). In the E-MTAB-1980 cohort, three pathway clusters were used by consensus matrices, and principal component analysis showed stable and robust clustering for the samples (Supplementary Figures S4A,B). Consensus clustering was then performed based on the above 39 differential pathways to divide the RCC patients in the TCGA-KIRC cohort into three clusters (Cluster A, Cluster B, and Cluster C) with distinct metabolic pathway profiles (Figure 3D). Patients in the three clusters experienced different clinical outcomes, and the OS of Cluster C was significantly higher than the OS of the other subtypes (Figure 3E). PFS showed a similar trend (Figure 3F).

### Quantification of the Tumour Microenvironment and Metabolic State in RCC by MRPScore

**FIGURE 4:**
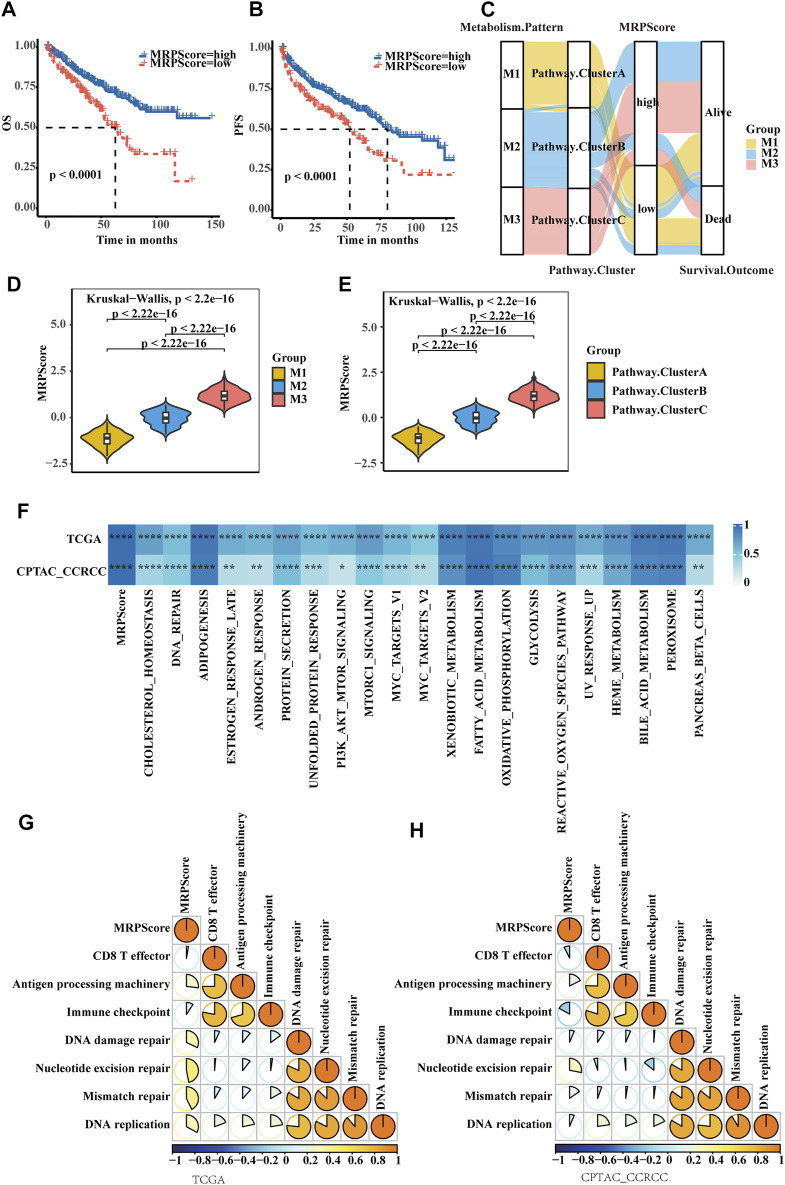
Quantification of metabolic signatures based on the MRPScore in the TCGA-KIRC cohort. **(A)** OS analyses for high- and low-MRPScore patient groups in the TCGA-KIRC cohort using Kaplan-Meier curves. **(B)** PFS survival analyses for high- and low-MRPScore patient groups in the TCGA-KIRC cohort using Kaplan-Meier curves. **(C)** Alluvial diagram of the metabolic patterns in groups with different pathway clusters, MRPScores, and survival outcomes. **(D)** Differences in the MRPScores among the three metabolic patterns in the TCGA-KIRC cohort. The Kruskal–Wallis test was used to compare the significant differences between the three metabolic patterns. **(E)** Differences in the MRPScores among the three pathway clusters in the TCGA-KIRC cohort. The Kruskal–Wallis test was used to compare the significant differences between the three metabolic patterns. **(F)** Correlations between the MRPScore and the known HALLMARK gene sets in the TCGA-KIRC and CPTAC-ccRCC cohorts using Pearson analysis T. **(G,H)** Correlations between the MRPScore and the known gene signatures in the TCGA-KIRC **(G)** and CPTAC-ccRCC **(H)** cohorts using Pearson analysis. A negative correlation is marked with blue, and a positive correlation is marked with orange. The asterisks represent a statistically significant *p* value (*p < 0.05; **p < 0.01; ***p < 0.001; ****p < 0.0001).

To make these RCC subtypes defined by metabolism-related gene signatures usable in clinical practice, we defined a scoring system named the MRPScore to quantify the metabolic status of each RCC patient. We found that the high-MRPScore group showed prominent survival and PFS benefits, while the low-MRPScore group exhibited much poorer survival and PFS (Figures 4A,B). The prognostic value of the MRPScore was then validated in the E-MTAB-1980 cohorts, where the high-MRPScore group also had an improved OS (Supplementary Figure S4C). An alluvial diagram was used to visualize the changes in the attributes of patients. Consistent with the above findings, a high MRPScore was linked with better survival, and a low MRPScore was mostly composed of the Cluster A subtype and M1 pattern (Figure 4C; Supplementary Figure S4D).

Then, we compared the differences in MRPScore among the three metabolic patterns and three pathway clusters in the TCGA-KIRC cohort. We found that the M1 group and Cluster A had low MRPScores and that the M3 group and Cluster C had high MRPScores (Figures 4D,E). For the E-MTAB-1980 cohort, the correlations among the metabolic patterns, metabolic pathways and MRPScore were the same as those in the TCGA-KIRC cohort (Supplementary Figures S4E,F). Pearson analysis showed that the MRPScore was strongly correlated with the known hallmark gene sets in the TCGA-KIRC, CPTAC-ccRCC and E-MTAB-1980 cohorts. (Figure 4F; Supplementary Figure S4G). Pearson analysis showed that the MRPScore was positively correlated with antigen processing machinery, nucleotide excision repair and mismatch repair and negatively correlated with CD8^+^ T effectors and immune checkpoints in the known gene signatures of the TCGA-KIRC and CPTAC-ccRCC cohorts (Figures 4G,H).

### Association Between the MRPScore and Clinical Characteristics of RCC

**FIGURE 5:**
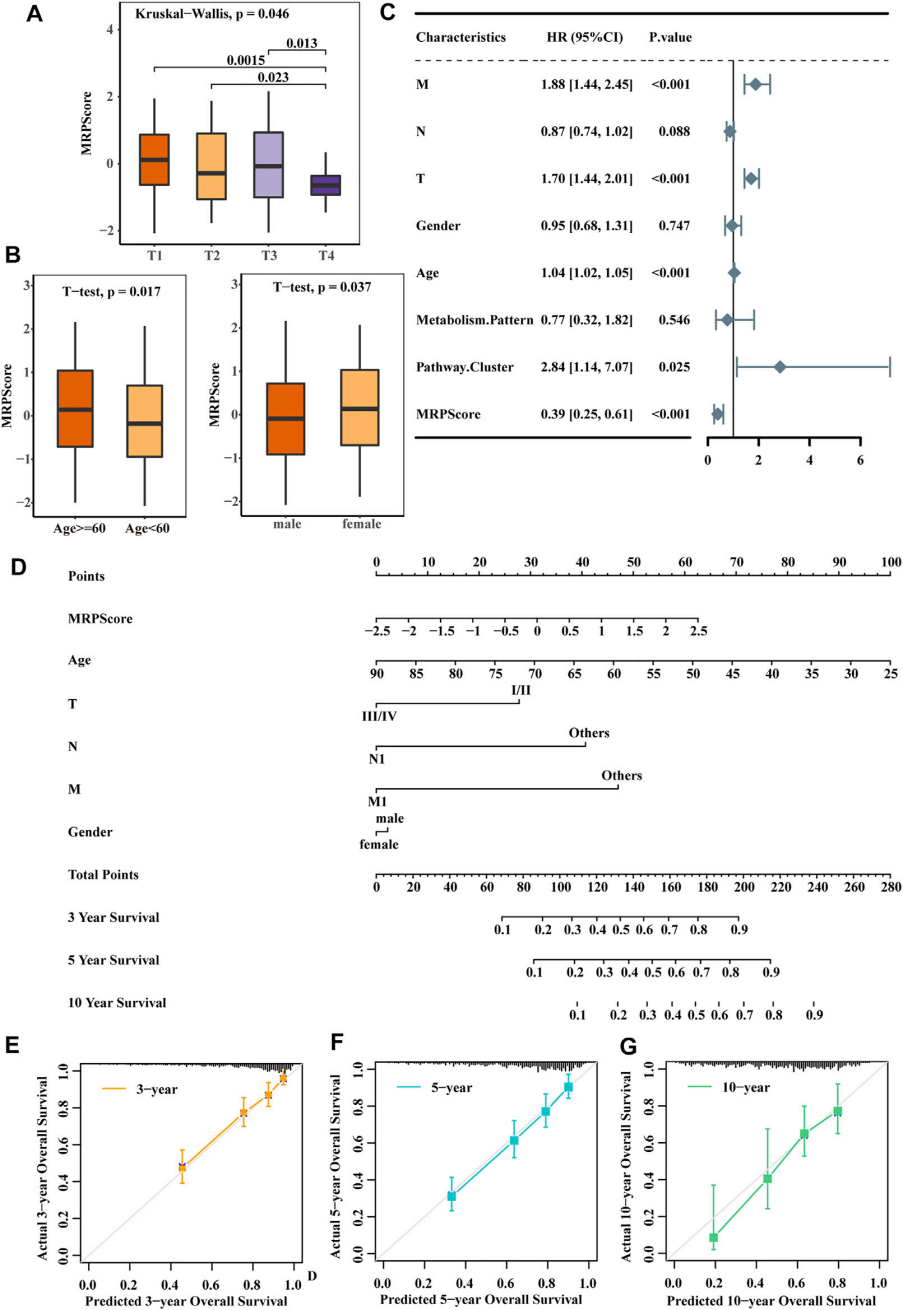
The MRPScore acted as a risk factor for RCC. **(A)** Differences in the MRPScores between the different T stages. The Kruskal–Wallis test was used to compare the significant differences between the 4T stages. The asterisks represent a statistically significant *p* value (*p < 0.05; **p < 0.01; ***p < 0.001; ****p < 0.0001). **(B)** Differences in the MRPScores between the different ages (left) and sexes (right). The *t* test was used to compare the significant differences. *p* values were determined by a student’s *t* test. **(C)** Multivariate Cox regression analysis results show the association between the clinicopathological parameters, such as age, sex, T stage (T), tumour lymph node (N), tumour metastasis (M), and MRPScore, of the new survival model and the OS of KIRC patients. **(D)** Nomogram construction for the 3-, 5- and 10-years OS prediction for KIRC patients. **(E–G)** Calibration curve analysis of the nomogram for predicting 3- **(E)**, 5- **(F)** and 10- **(G)** year OS in the TCGA-KIRC dataset.

After analysing the relationship between the clinical traits and MRPScore in the TCGA-KIRC cohort, we found that patients with a low MRPScore experienced a high T stage, and a high MRPScore was more associated with a low T stage (Figure 5A). This may be the reason why low MRPScores were associated with poor prognosis. In addition, the MRPScore was associated with age and sex (Figure 5B). Then, we analysed the correlation between the MRPScore and survival rate by multivariate Cox regression analysis and indicated that the MRPScore was an independent and robust prognostic factor for RCC (Figure 5C). OS nomogram models of 3-, 5- and 10-years OS were established (Figure 5D). Calibration curve analysis of the nomogram for predicting 3-, 5- and 10-years OS in the TCGA-KIRC dataset was performed (Figures 5E–G). We compared the association of OS (Supplementary Figures S5A,B) and PFS (Supplementary Figures S5C,D) with the MRPScore according to the T stage in the TCGA-KIRC cohort and showed that regardless of T stage, a high MRPScore was significantly associated with a better prognosis. This was also true in the CPTAC-CCRCC and TCGA-KIRP cohorts (Supplementary Figures S5E–L).

### MRPScore Predicts the Clinical Response to Immune Checkpoint Inhibitor Therapy

**FIGURE 6:**
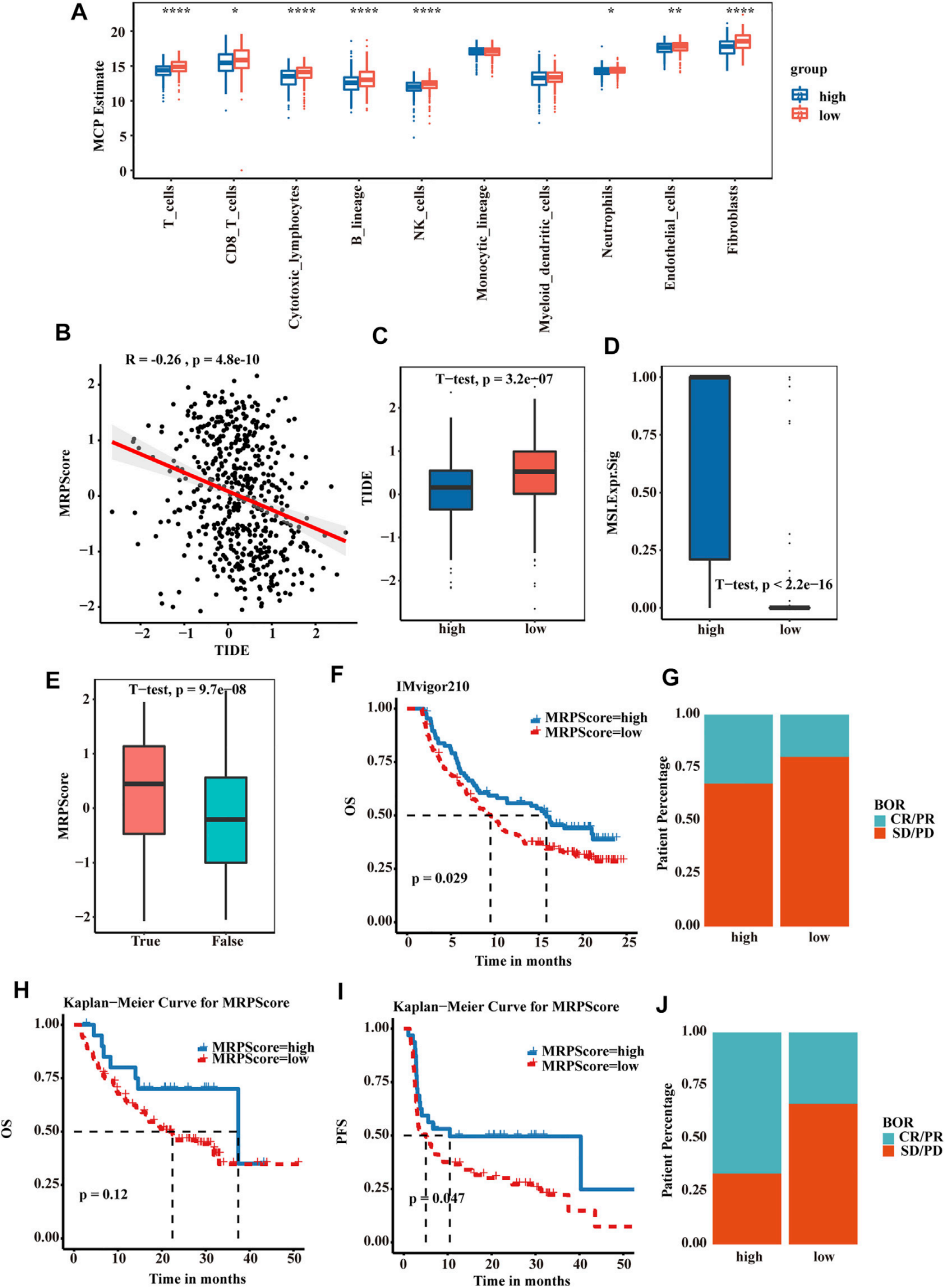
MRPScore as an indicator for predicting the response to immunotherapy. **(A)** Boxplot of the abundance of immune and stromal cell populations distinguished by the high- and low-MRPScore groups. **(B)** Scatter plots showing the significant negative correlations between the MRPScore and TIDE score in the TCGA-KIRC cohort. **(C)** Boxplot representing significantly higher MRPScores in the low-MRPScore group. **(D)** Boxplot representing a significantly higher MSI expression signature in the high-MRPScore group. **(E)** Boxplot showing significantly higher MRPScores for responders in the TCGA-KIRC dataset. **(F)** Survival analyses for high- and low-MRPScore patient groups in the anti-PD-L1 immunotherapy cohort using Kaplan–Meier curves (IMvigor210 cohort). **(G)** The proportion of patients who responded to PD-L1 blockade immunotherapy in the high- or low-MRPScore groups (IMvigor210 cohort). SD, stable disease; PD, progressive disease; CR, complete response; PR, partial response. **(H,I)** Kaplan-Meier curve representing OS **(H)** and PFS **(I)** for the high-MRPScore and low-MRPScore groups in the melanoma cohort. **(J)** The proportion of patients who responded to immune checkpoint blockade therapy in the high-MRPScore and low-MRPScore groups in the melanoma cohort. p values were determined by a student’s *t* test.

We then explored the relationship between the MRPScore and immune cells and stromal cells. In the boxplot, the low MRPScore group had high T cells, B cells, NK cells and fibroblasts (Figure 6A). Immunologic checkpoint inhibitors (ICIs) that block the T cell inhibitory molecules programmed death-1 receptor (PD-1) and programmed death-1 ligand (PD-L1) are an emerging anticancer treatment with improved survival benefits (56). The tumour immune dysfunction and exclusion (TIDE) algorithm is a model that estimates the potential response to ICI therapy. Therefore, by using the TIDE algorithm, we explored whether the MRPScore could evaluate the responses to ICI therapy in TCGA-KIRC cohorts for the estimation of ICI therapy efficacy. As a result, we found a significant negative correlation between the MRPScore and TIDE score in the cohort, and a high MRPScore was associated with a lower TIDE score (Figures 6B,C). Mismatch repair deficiency (dMMR) results in microsatellite instability (MSI) and is strongly associated with responsiveness to PD-1 blocking antibodies. MSI-high tumours have significantly higher sensitivity to ICIs than patients with MSI-low tumours and have derived clinical benefits from immunotherapy. We found that the MSI expression signature was significantly higher in the high-MRPScore group than in the low-MRPScore group (Figure 6D). In the TCGA-KIRC dataset, the MRPScores were significantly higher for the immunotherapy responders (Figure 6E). The same results were observed in the E-MTAB-1980 cohort (Supplementary Figures S6A–D) and in the CPTAC-CCRCC cohort (Supplementary Figures S6E–H). For IMvigor210, which is an anti-PD-L1 immunotherapy cohort, the survival analyses showed that high-MRPScore patients had a better prognosis, while the low-MRPScore group had poor survival (Figure 6F). Patients with a high MRPScore had a higher proportion of complete response or partial response to PD-L1 blockade immunotherapy, which may be more beneficial for immunotherapy (Figure 6G). This could be validated in the metastatic melanoma (Figures 6H–J) and GSE78220 cohorts (Supplementary Figures S6I–K).

## Discussion

Renal cell carcinoma is a common malignancy of the urinary system, and it is an aggressive disease state that is insensitive to radiation. It has a poor prognosis and lacks effective therapeutic options. Previous studies have revealed that metabolic dysfunction plays an important role in the development of most cancers, including prostate cancer, (Sreekumar et al., 2009), breast cancer, (Jaramillo and Zhang, 2013), brain cancer (Prabhu et al., 2014) and liver cancer. (Budhu et al., 2013; Huang et al., 2013). In RCC, many studies have revealed that classical metabolic pathways are increased, decreased, or bypassed entirely in the development of cancer. Many metabolic pathways, such as Warburg metabolism, are reprogrammed in this disease, which has been confirmed to be a key component of ccRCC metabolic reprogramming (Perroud et al., 2006; Chen et al., 2016). Dysfunction of fatty acid oxidation and lipid synthesis are characteristics of ccRCC (Horiguchi et al., 2008; Ganti et al., 2012). Tryptophan metabolism is reduced, leading to immunosuppression (Fallarino et al., 2002). The increase in glutamine metabolism is involved in the development of ccRCC (Wettersten et al., 2015; Hakimi et al., 2016). However, the cause of cancer is not only led by a specific dysfunction of one metabolic pathway but also an overall metabolic change that requires comprehensive analysis. In this study, we integrated metabolism-related genes and metabolism-related pathways to predict the prognosis of RCC. We obtained three metabolic clusters and three metabolic patterns that were associated with different OS and PFS rates. Those clusters and patterns were related to different metabolic statuses. The M3 pattern and Cluster C were metabolism active, the M1 pattern and Cluster A were metabolism silent, and the M2 pattern and Cluster B were moderately active. Interestingly, M1 correlated with poor prognosis but had more T cells, cytotoxic lymphocytes, NK cells and fibroblasts, which may be because global metabolism silencing causes a low metabolism level of effector T cells. This recruits more lymphocytes but does not kill cancer cells. Recently, many studies have shown that reduced glycolysis in CD8^+^ T effector cells inhibits their activity, including antitumour effects (Chang et al., 2015; Gemta et al., 2019; Hu et al., 2019). However, more fibroblasts may prevent immune cells from infiltrating into tumour tissue even when immune cells are enriched. In a previous study, stromal activation in the TME was considered to be immunosuppressive, which is consistent with our hypothesis (Mariathasan et al., 2018).

During the past decade, RCC treatment has transitioned from a nonspecific immune approach to targeted therapy against vascular endothelial growth factor (VEGF) and now to novel immunotherapy agents (Siegel et al., 2016; Choueiri and Motzer, 2017). However, the immunotherapeutic responses to ICIs are variable among RCC patients. Some patients achieve complete remission, and other patients show continuous progression. Hence, it is urgent to establish a reliable tool for the appropriate selection of immunotherapies for patients in clinical practice. In this study, we established a scoring evaluation system named the MRPScore, which is a robust metabolism classifier for classifying RCC patients with different responses to immunotherapy. We determined the MRPScore to quantify the metabolic activation status. The high-MRPScore group was considered to have metabolic activation and demonstrated a significant correlation with immune checkpoints. A low-MRPScore was associated with poor prognosis and worse subtype. Moreover, we demonstrated that there were significantly higher MRPScores for immunotherapy responders than for patients in the low-MRPScore group. In short, the MRPScore could be used to comprehensively evaluate the metabolic pattern and corresponding TME infiltration characteristics of individual patients to further determine the immune phenotypes of tumours and guide clinical practice. The MRPScore could be used to evaluate the cellular, molecular and genetic factors associated with tumour inflammation, tumour differentiation levels, and response to immunotherapy. The MRPScore could act as an independent prognostic marker to predict patient survival. A high MRPScore implied increased sensitivity to ICIs. This indicates that the application of the MRPScore could help personalize the treatment of RCC patients and assist in making decisions for clinical practice.

In brief, our analysis indicates that the MRPScore is an independent risk factor for RCC, thereby providing an ideal predictor for the prognosis and therapeutic response of RCC patients. The limitations of this study were that the stability of the MRPScore was tested through the cross validation of six cohorts, but the signature will be more reliable if it is tested by prospective cohort studies in the future. The results of single-cell sequencing should be able to explain the specific changes in the tumour microenvironment, which is also an aspect of our attention in the future. Moreover, our model should be validated further by performing both *in vitro* and *in vivo* experiments to better evaluate the relationship between the MRPScore and immune cells.

## Data Availability

The original contributions presented in the study are included in the article/Supplementary Material, further inquiries can be directed to the corresponding authors.
