# Supplementary figures and images for: Metabolism-Related Signature Analysis Uncovers the Prognostic and Immunotherapeutic Characteristics of Renal Cell Carcinoma
Source: Front Mol Biosci. 2022 Mar 28;9:837145. doi: 10.3389/fmolb.2022.837145 (PMC8995851; doi:10.3389/fmolb.2022.837145)

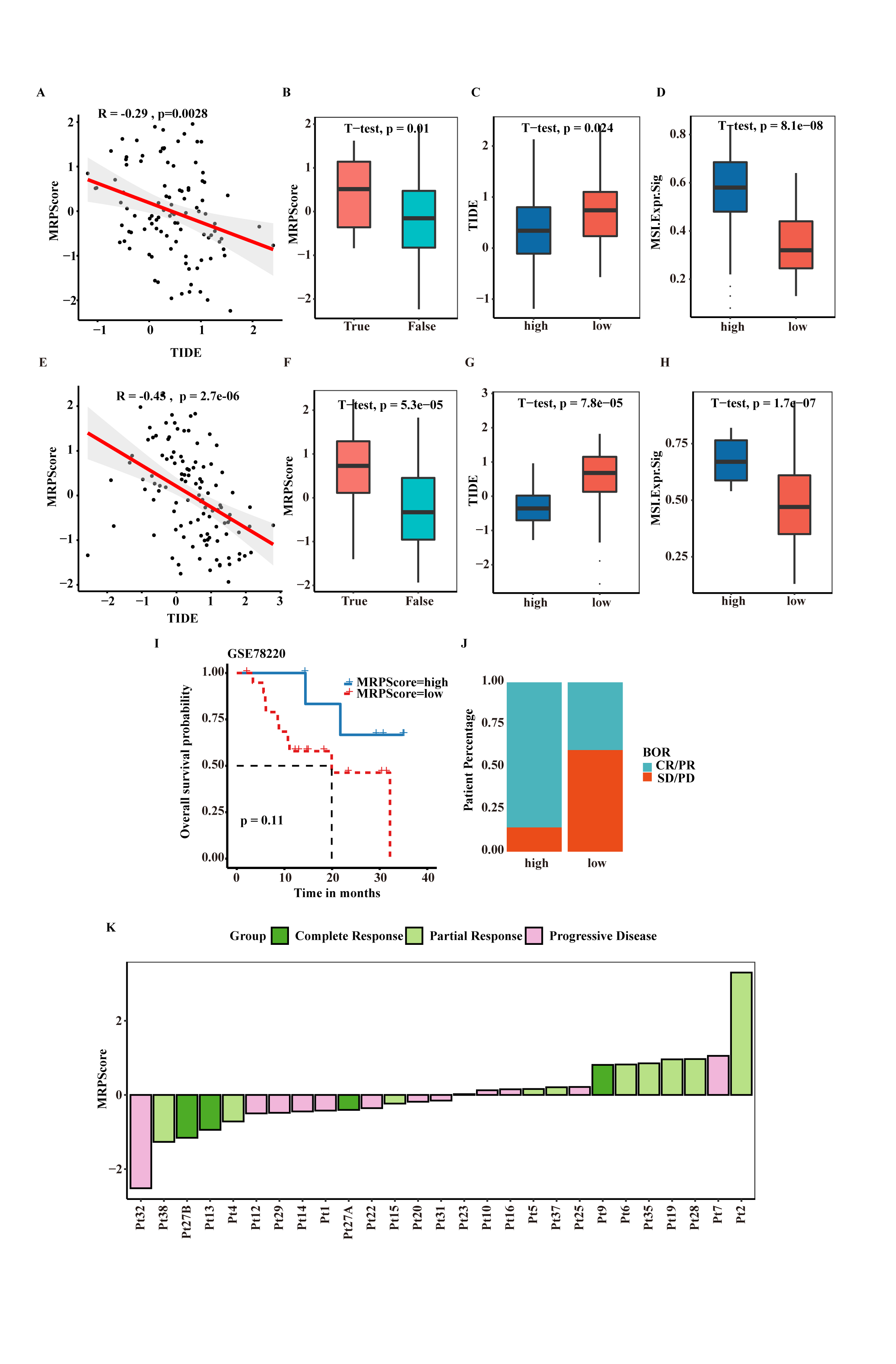

Supplement: Supplementary file 1 [file Image6.TIF]

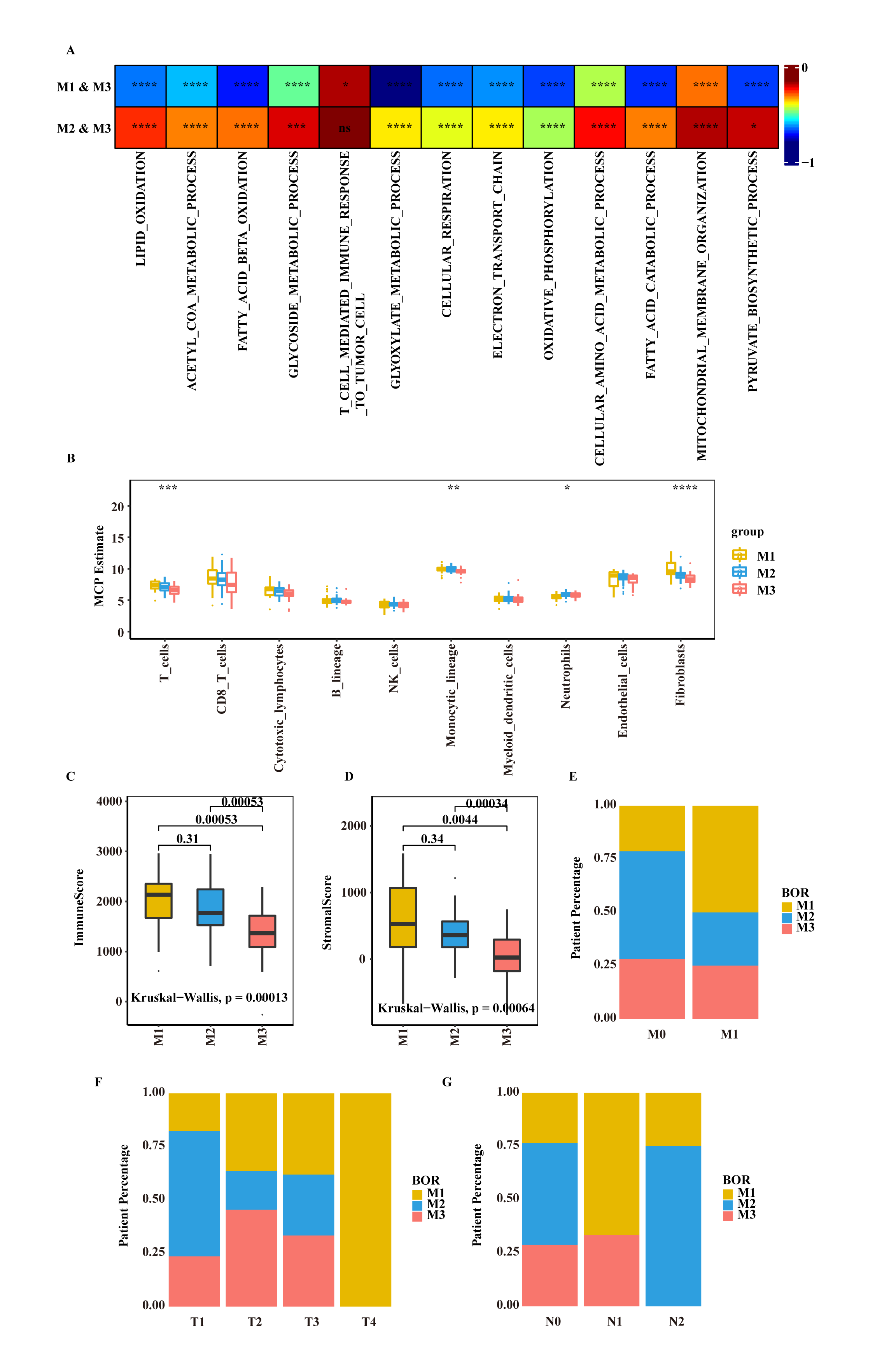

Supplement: Supplementary file 2 [file Image3.TIF]

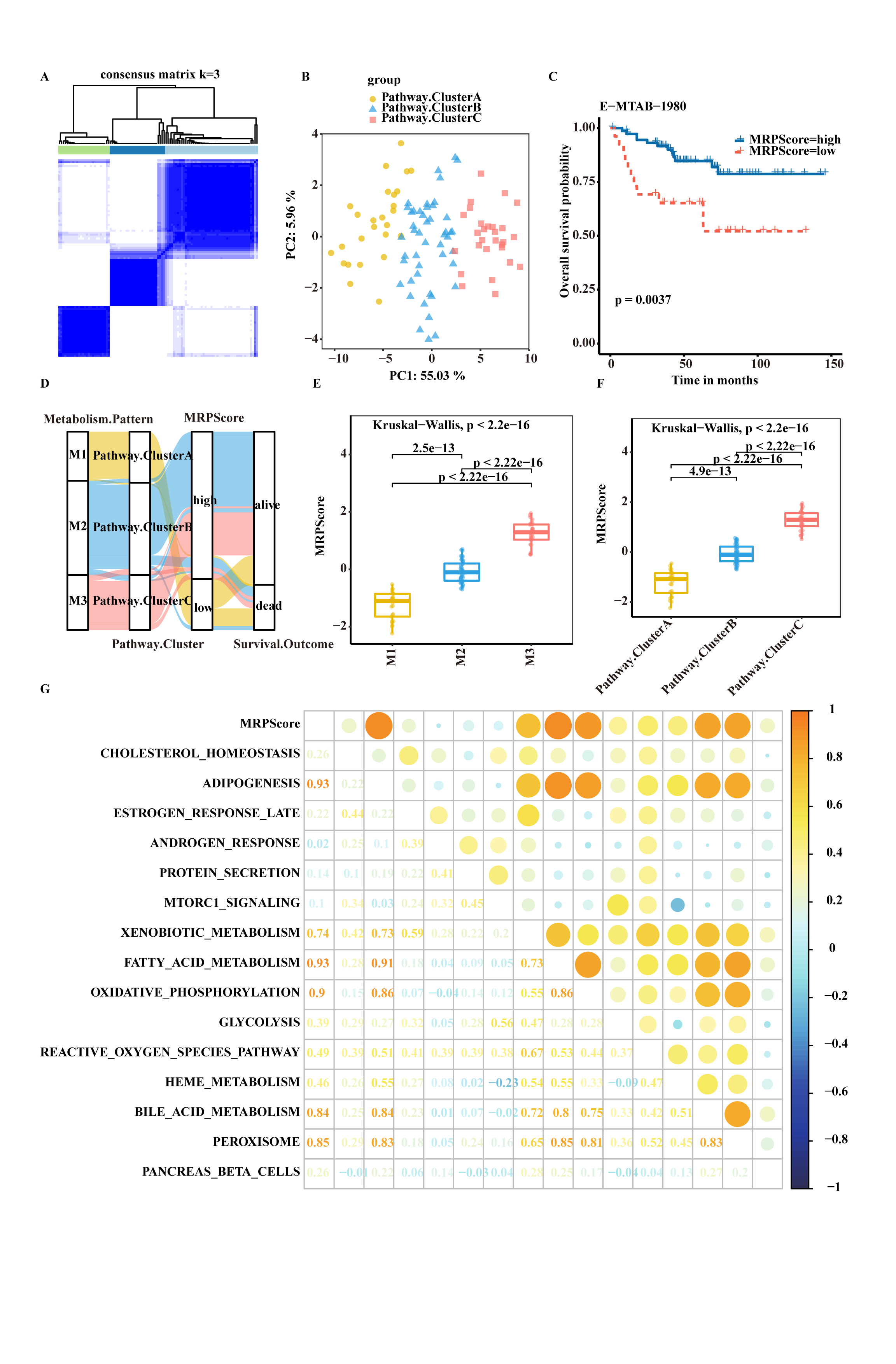

Supplement: Supplementary file 3 [file Image4.TIF]

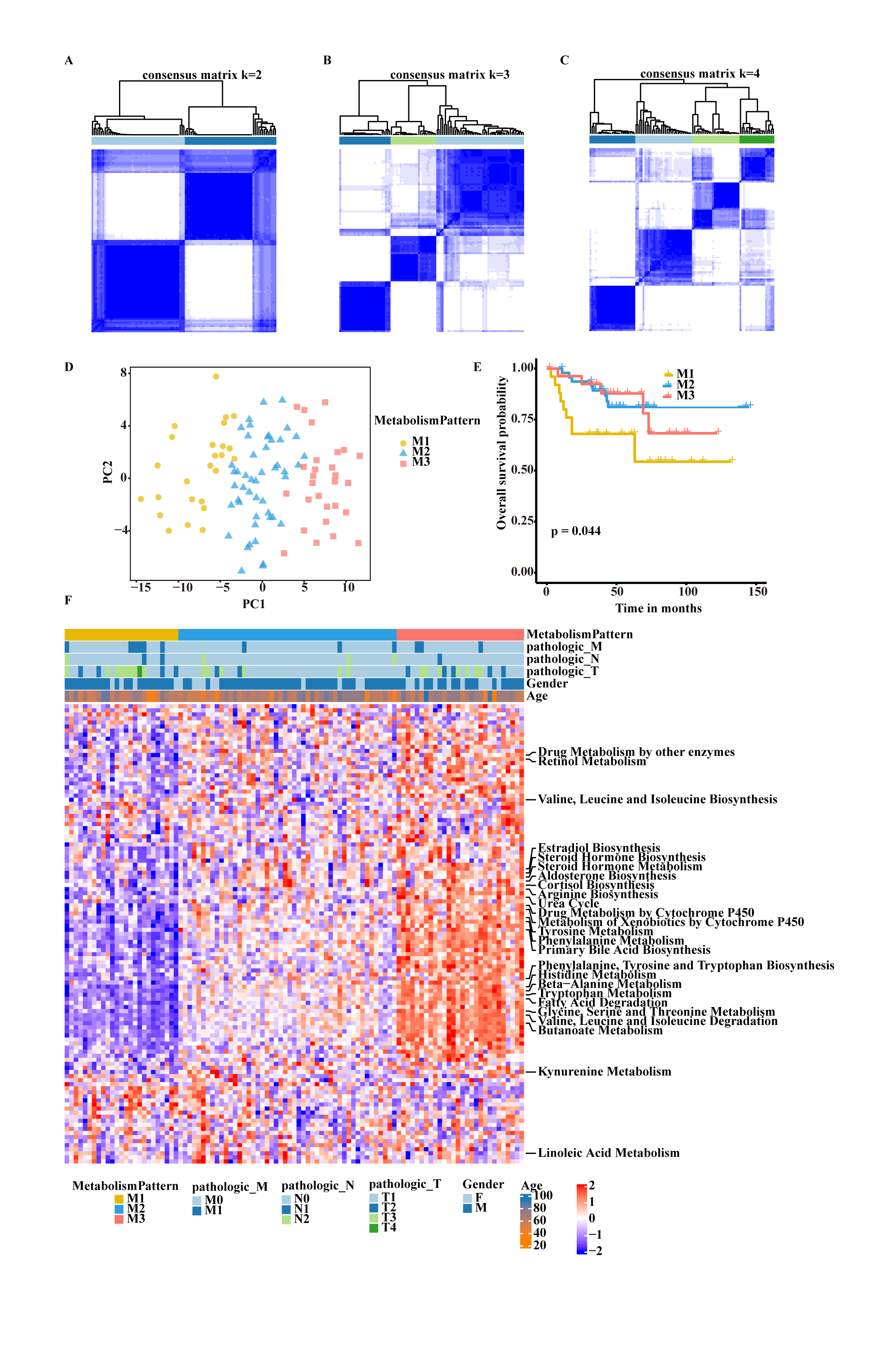

Supplement: Supplementary file 4 [file Image2.TIF]

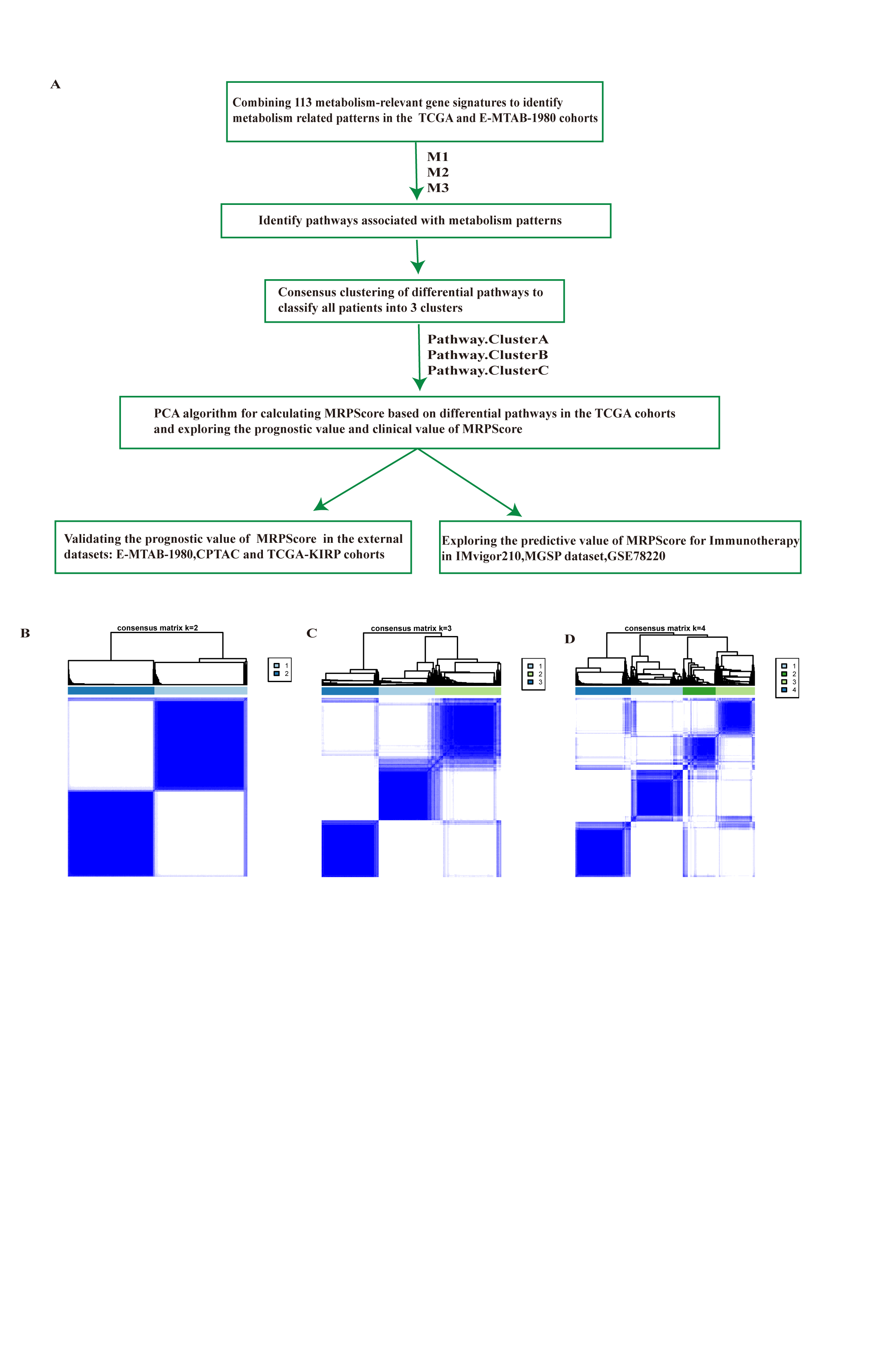

Supplement: Supplementary file 5 [file Image1.TIF]

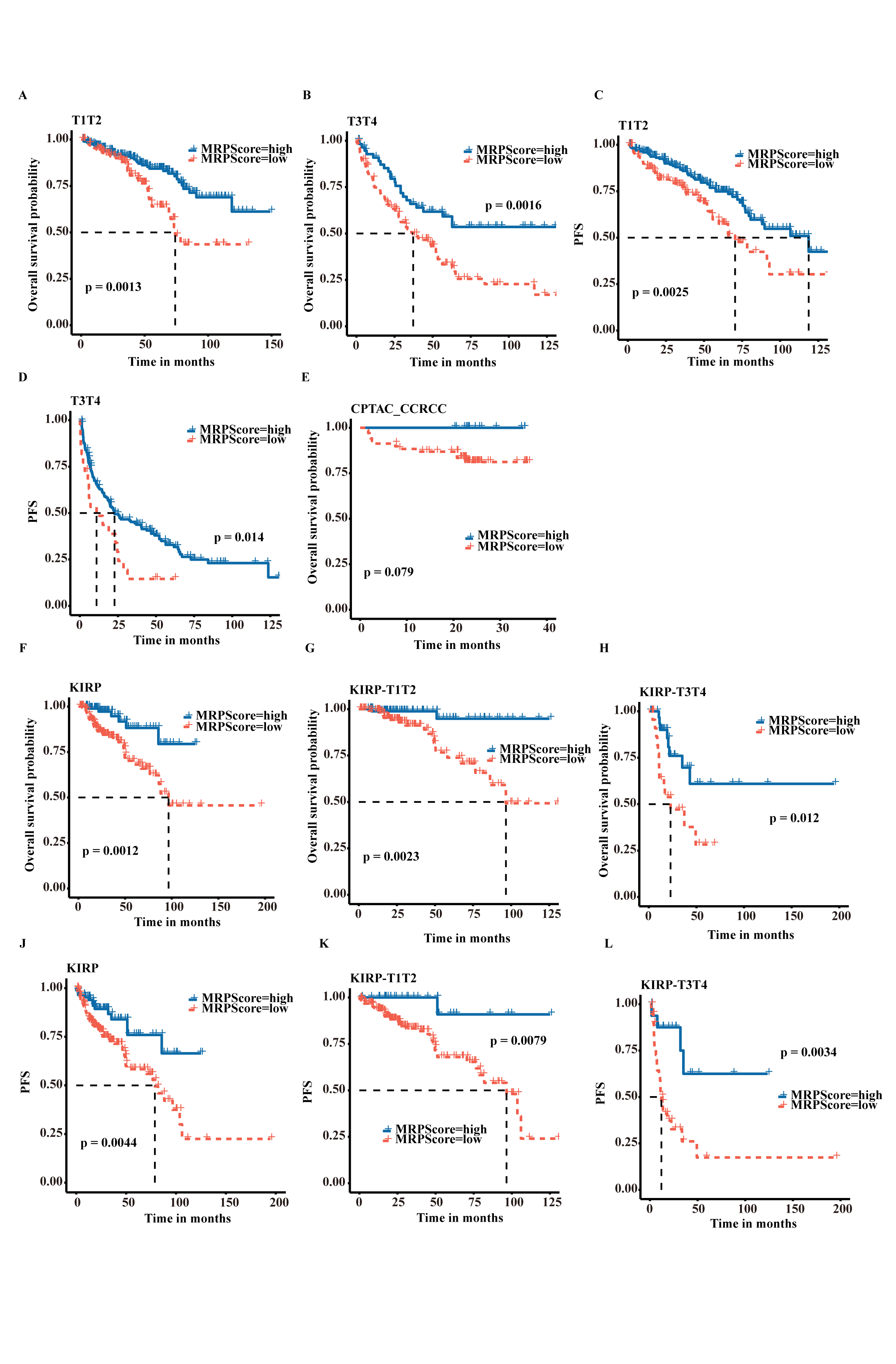

Supplement: Supplementary file 6 [file Image5.TIF]
